# Healthcare workers in Singapore infected with COVID‐19: 23 January‐17 April 2020

**DOI:** 10.1111/irv.12803

**Published:** 2020-09-13

**Authors:** Lai Yin Wong, Aidan Lyanzhiang Tan, Yee‐Sin Leo, Vernon Jian Ming Lee, Matthias Paul Han Sim Toh

**Affiliations:** ^1^ Chronic Disease Epidemiology, Population Health National Healthcare Group Singapore; ^2^ Health Services and Outcomes Research National Healthcare Group Singapore; ^3^ National Centre for Infectious Diseases Singapore; ^4^ Tan Tock Seng Hospital Singapore; ^5^ Saw Swee Hock School of Public Health Singapore; ^6^ Lee Kong Chian School of Medicine Singapore; ^7^ Yong Loo Lin School of Medicine Singapore; ^8^ Ministry of Health Singapore Singapore

**Keywords:** characteristic, COVID‐19, exposure, healthcare worker, occupation

## Abstract

**Objective:**

To describe the characteristics of healthcare workers (HCWs) infected with COVID‐19 and to examine their sources of exposure.

**Methods:**

A descriptive cross‐sectional study using data extracted from the centralized disease notification system comprising individuals confirmed with COVID‐19 in Singapore between 23 January and 17 April 2020. Occupation of HCWs was categorized into six categories. Their job nature was classified into “frontline” or “back‐end” based on the frequency of direct patient contact, and source of exposure was classified as family/household, social interaction or workplace. Chi‐square and median tests were used to identify differences between categorical groups and sample medians, respectively.

**Results:**

A total of 88 (1.7%) HCWs were identified from 5,050 cases. Their median age was 35 years. Chinese and Indians constituted 42.0% and 31.8%, respectively, and 43.2% were foreigners. The majority (63.6%) was serving at frontlines handling patient‐facing duties, 15.9% were doctors, 11.4% were nurses and 44.3% were ancillary staff. About 81.8% acquired the infection locally, of which 40.3% did not have a clearly identifiable source of exposure. Exposure from the family/household was most common (27.8%), followed by workplace (16.7%) and social interaction (15.3%). All HCWs were discharged well with no mortality; three (3.4%) were ever admitted to intensive care unit and required increased care.

**Conclusion:**

Healthcare workers accounted for a small proportion of COVID‐19 cases in Singapore with favourable outcomes. The possibility of transmission resulting from family/household exposure and social interactions highlights the need to maintain strict vigilance and precautionary measures at all times beyond the workplace.

## 1 INTRODUCTION

Since the first case of coronavirus disease 2019 (COVID‐19) was detected in China in December 2019, the disease has spread rapidly worldwide with an unprecedented scale of impact. The infective agent, severe acute respiratory syndrome coronavirus 2 (SARS‐CoV‐2), is highly contagious. One infected person may subsequently lead up to 5.7 confirmed cases.^1^ Its high transmissibility has resulted in many infections and hospitalizations, even among healthcare workers (HCWs).^2^ Although the case fatality rate of COVID‐19 is lower than that of severe acute respiratory syndrome (SARS) (9.6%) that resulted in 8,096 cases worldwide during the eight‐month outbreak in 2003,^3^ COVID‐19 has significantly more absolute number of fatalities. By 1 June 2020, 6.2 million of COVID‐19 cases had been detected globally with case fatality rate of 6.0%.^4^ Its death toll in China was nearly three times as many people in eight weeks than that of SARS in eight months.^5^

Healthcare workers are defined as paid or unpaid persons engaged in actions whose primary intent is to enhance health.^6^ HCWs are at higher risk^7^ of acquiring COVID‐19 due to increased occupational exposure to SARS‐CoV‐2. In China, nearly 4% of the confirmed cases in Wuhan^8^ during the initial phases of pandemic were among HCWs, due primarily to inadequate protection measures in clinical departments and shortage of personal protective equipment (PPE).^9^, ^10^ In Singapore, prior to detecting the first COVID‐19 case on 23 January 2020, hospitals had already enforced the use of PPE and enhanced fever and sickness surveillance among frontline staff to protect and monitor potentially exposed HCWs.^11^ Singapore reported the infection of its first HCW with COVID‐19 on 13 February. Infected HCWs are at risk of transmitting disease to vulnerable patients under their care. Knowing the details of job nature of HCWs and potential exposure to COVID‐19 is crucial for risk management and prioritizing workplace crisis response plans. Healthcare institutions can identify gaps in upstream preventive measures at the workplace such as adequacy in training to handle emerging infectious diseases or adherence to standard operating procedures.

This study seeks to examine the characteristics of HCWs in Singapore who were detected to have SARS‐CoV‐2 between 23 January and 17 April 2020 and identify their source of exposure to prevent future infections.

## 2 METHODOLOGIES

### 2.1 Case confirmation

Methodologies employed in case confirmation were described previously.^12^ From 23 January to 17 April 2020, individuals suspected of COVID‐19 infection were tested for the presence of SARS‐CoV‐2 RNA using laboratory‐based polymerase chain reaction test. Confirmed cases nationwide were notified to the Ministry of Health Singapore by attending physicians or laboratories. Every confirmed case undergoes detailed epidemiological investigations and is interviewed by the trained personnel collecting data on socio‐demographic profile, visits to physician, dates of exposure to other confirmed COVID‐19 case and onset of symptoms, and daily activities up to 14 days prior to symptom onset. For cases who worked, details of occupation, their workplace environment and contact with colleagues were obtained.

### 2.2 Identifying healthcare workers

This study adopted the approach employed by the World Health Organization^6^ to define HCWs. All paid workers engaged by the healthcare institutions or those whose personal actions are primarily intended to improve health but who work for non‐healthcare institutions were included.^6^

To better characterize HCWs, information on occupation was classified into six job categories encompassing doctor, nurse, allied health, ancillary staff, administrative staff and construction worker. Their nature of work was broadly classified into “frontline” or “back‐end” based on frequency of direct patient contact required. Frontline refers to duties that directly interacted with patients, whilst back‐end refers to non‐clinical duties with minimal to no patient contact.

### 2.3 Classifying sources of infection and exposure

To ascertain the source of infection among HCWs, their exposures as collected by the contact tracing interviews were reviewed. Cases with overseas travel to countries with higher incidence of cases compared to Singapore within the past 14 days were considered imported. Cases without relevant travel history were considered locally acquired. Among the locally acquired cases, those with documented local exposure sources were classified as related to family/household, social interaction or the workplace, based on the relationships between the probable infector and infectee, where the patient with earlier symptom onset date being identified as the probable infector. In this study, workplace exposure included both occupational exposure between HCWs and patients, and exposure to other infected HCWs within the workplace. The exposure source was classified as unidentified if the probable infector could not be determined.

### 2.4 Data analysis

This is a descriptive cross‐sectional study using data extracted from the centralized disease notification system. Categorical variables were analysed using number and proportions, whilst continuous measurements were described using median values. Where appropriate, between‐group comparisons were conducted using chi‐square and median tests to identify differences between categorical groups and sample medians, respectively, where probability less than 0.05 was considered statistically significant. Statistical Packages for Social Sciences (SPSS) v22.0 (IBM) was used for data analysis.

## 3 RESULTS

Of the 5,050 COVID‐19 cases reported nationwide between 23 January and 17 April 2020, 1.7% were identified as HCWs. The median age of HCWs was 35 years (IQR: 27‐50 years), 54.5% were female, and 42.0% and 31.8% were of Chinese and Indian ethnicities, respectively (Table 1). About 43.2% of the HCWs were foreigners, predominantly from India and Malaysia. Foreign HCWs were significantly younger than the local counterparts (median ages: 32 vs 40 years; *P* = .004), and they made up 53.8% of those below 50 years.

**TABLE 1:** Demographic profile of healthcare workers with COVID‐19, 23 January‐17 April 2020

Socio‐demographic profile	No.	%
Total	**88**	**100.0**
Age group (y)		
Below 30	29	33.0
30‐39	25	28.4
40‐49	11	12.5
50‐59	14	15.9
60‐69	9	10.2
Gender		
Male	40	45.5
Female	48	54.5
Ethnic group		
Chinese	37	42.0
Malay	9	10.2
Indian	28	31.8
Others	14	15.9
Nationality		
Singapore	50	56.8
India	17	19.3
Malaysia	9	10.2
Philippines	3	3.4
Bangladesh	5	5.7
Others^a^	4	4.5
Job nature		
Frontline	56	63.6
Back‐end	32	36.4
Workplace		
Public sector	60	68.2
Private sector	28	31.8
Job category		
Doctor	14	15.9
Nurse	10	11.4
Allied health professional	12	13.6
Ancillary staff	39	44.3
Administrative staff	8	9.1
Construction worker	5	5.7
Department of doctors and nurses working in hospitals (n = 14^b^)		
Emergency department	2	14.3
Anaesthesia	2	14.3
Operating theatre	2	14.3
Ophthalmology	2	14.3
Infectious disease	1	7.1
Orthopaedic	1	7.1
Paediatrics	1	7.1
Dialysis centre	1	7.1
Rehabilitation sub‐acute ward	1	7.1
Radiology	1	7.1
Source of infection		
Imported	16	18.2
Local	72	81.8

^a^ Included Sri Lanka (2), China (1) and Germany (1).

^b^ Excluded missing value (3).

About 68.2% of the HCWs worked in the public sector. Of these, 83.3% worked in hospitals, 11.7% at national specialty centres and 5.0% at primary care polyclinics (Table 2). Among those who worked in the private sector, 28.6% worked in hospitals, 25.0% at primary care clinics, 21.4% at community services and the remaining 25.0% at other non‐healthcare–related institutions (Table 3). Overall, 63.6% served at the frontlines, with higher proportion in the private compared with public institutions (71.4% vs 60.0%).

**TABLE 2:** Healthcare workers with COVID‐19 by types of public institutions

Job category	Public sector						Total	
	Tertiary hospital		National specialty centre		Polyclinic			
	No.	%	No.	%	No.	%	No.	%
Total	**50**	**100.0**	**7**	**100.0**	**3**	**100.0**	**60**	**100.0**
Frontline/frequent patient contact	**27**	**54.0**	**6**	**85.7**	**3**	**100.0**	**36**	**60.0**
Doctor	6	12.0	3	42.9	0	0.0	9	15.0
Nurse	7	14.0	0	0.0	0	0.0	7	11.7
Allied health	5	10.0	0	0.0	0	0.0	5	8.3
Medical social worker	*2*	*4.0*	*0*	*0.0*	*0*	*0.0*	*2*	*3.3*
Physiotherapist/speech therapist	*2*	*4.0*	*0*	*0.0*	*0*	*0.0*	*2*	*3.3*
Psychologist/psychotherapist	*1*	*2.0*	*0*	*0.0*	*0*	*0.0*	*1*	*1.7*
Ancillary staff	9	18.0	3	42.9	3	100.0	15	25.0
Healthcare assistant/attendant	*2*	*4.0*	*0*	*0.0*	*2*	*66.7*	*4*	*6.7*
Porter	*3*	*6.0*	*1*	*14.3*	*0*	*0.0*	*4*	*6.7*
Patient service associate	*0*	*0.0*	*1*	*14.3*	*1*	*33.3*	*2*	*3.3*
Coordinator	*2*	*4.0*	*0*	*0.0*	*0*	*0.0*	*2*	*3.3*
Dental assistant	*0*	*0.0*	*1*	*14.3*	*0*	*0.0*	*1*	*1.7*
Patient therapy associate	*1*	*2.0*	*0*	*0.0*	*0*	*0.0*	*1*	*1.7*
Clinical specialist	*1*	*2.0*	*0*	*0.0*	*0*	*0.0*	*1*	*1.7*
Back‐end/minimal patient contact	**23**	**46.0**	**1**	**14.3**	**0**	**0.0**	**24**	**40.0**
Administrative staff	3	6.0	0	0.0	0	0.0	3	5.0
Administrator	*2*	*4.0*	*0*	*0.0*	*0*	*0.0*	*2*	*3.3*
Manager	*1*	*2.0*	*0*	*0.0*	*0*	*0.0*	*1*	*1.7*
Ancillary staff	15	30.0	1	14.3	0	0.0	16	26.7
Technician	*8*	*16.0*	*1*	*14.3*	*0*	*0.0*	*9*	*15.0*
Maintenance worker	*4*	*8.0*	*0*	*0.0*	*0*	*0.0*	*4*	*6.7*
Cleaner or housekeeper	*2*	*4.0*	*0*	*0.0*	*0*	*0.0*	*2*	*3.3*
Painter	*1*	*2.0*	*0*	*0.0*	*0*	*0.0*	*1*	*1.7*
Construction worker	5	10.0	0	0.0	0	0.0	5	8.3

**TABLE 3:** Healthcare workers with COVID‐19 by types of private institutions

Job category	Private sector								Total	
	Private hospital		Private practice or general practitioner		Community service^a^		Others^b^			
	No.	%	No.	%	No.	%	No.	%	No.	%
Total	**8**	**100.0**	**7**	**100.0**	**6**	**100.0**	**7**	**100.0**	**28**	**100.0**
Frontline/frequent patient contact	**5**	**62.5**	**7**	**100**	**4**	**66.7**	**4**	**57.1**	**20**	**71.4**
Doctor	2	25.0	2	28.6	0	0.0	1	14.3	5	17.9
Nurse	2	25.0	0	0.0	1	16.7	0	0.0	3	10.7
Allied health	0	0.0	2	28.6	2	33.3	3	42.9	7	25.0
Traditional Chinese Medicine physician	*0*	*0.0*	*0*	*0.0*	*1*	*16.7*	*1*	*14.3*	*2*	*7.1*
Medical social worker	*0*	*0.0*	*0*	*0.0*	*1*	*16.7*	*0*	*0.0*	*1*	*3.6*
Physiotherapist/speech therapist	*0*	*0.0*	*0*	*0.0*	*0*	*0.0*	*1*	*14.3*	*1*	*3.6*
Psychologist/psychotherapist	*0*	*0.0*	*1*	*14.3*	*0*	*0.0*	*0*	*0.0*	*1*	*3.6*
Radiographer	*0*	*0.0*	*1*	*14.3*	*0*	*0.0*	*0*	*0.0*	*1*	*3.6*
Pharmacist	*0*	*0.0*	*0*	*0.0*	*0*	*0.0*	*1*	*14.3*	*1*	*3.6*
Ancillary staff	1	12.5	3	42.9	1	16.7	0	0.0	5	17.9
Clinic assistant	*0*	*0.0*	*2*	*28.6*	*0*	*0.0*	*0*	*0.0*	*2*	*7.1*
Nursing aide	*0*	*0.0*	*0*	*0.0*	*1*	*16.7*	*0*	*0.0*	*1*	*3.6*
Receptionist	*0*	*0.0*	*1*	*14.3*	*0*	*0.0*	*0*	*0.0*	*1*	*3.6*
Health screening executive	*1*	*12.5*	*0*	*0.0*	*0*	*0.0*	*0*	*0.0*	*1*	*3.6*
Back‐end/minimal patient contact	**3**	**37.5**	**0**	**0.0**	**2**	**33.3**	**3**	**42.9**	**8**	**28.6**
Administrative staff	3	37.5	0	0.0	0	0.0	2	28.6	5	17.9
Administrator	*1*	*12.5*	*0*	*0.0*	*0*	*0.0*	*0*	*0.0*	*1*	*3.6*
Manager	*1*	*12.5*	*0*	*0.0*	*0*	*0.0*	*0*	*0.0*	*1*	*3.6*
Director	*0*	*0.0*	*0*	*0.0*	*0*	*0.0*	*1*	*14.3*	*1*	*3.6*
Researcher	*0*	*0.0*	*0*	*0.0*	*0*	*0.0*	*1*	*14.3*	*1*	*3.6*
Information technology	*1*	*12.5*	*0*	*0.0*	*0*	*0.0*	*0*	*0.0*	*1*	*3.6*
Ancillary staff	0	0.0	0	0.0	2	33.3	1	14.3	3	10.7
Technician	*0*	*0.0*	*0*	*0.0*	*1*	*16.7*	*0*	*0.0*	*1*	*3.6*
Cleaner or housekeeper	*0*	*0.0*	*0*	*0.0*	*1*	*16.7*	*0*	*0.0*	*1*	*3.6*
Medicine despatcher	*0*	*0.0*	*0*	*0.0*	*0*	*0.0*	*1*	*14.3*	*1*	*3.6*

^a^ Incl. charitable organizations, old‐folks home or other organizations for the vulnerable groups.

^b^ Incl. non‐healthcare–related organizations (eg shopping mall, research company).

There were significantly more females than males (85.4% vs 37.5%; *P* < .001) and more local residents than foreigners (78.0% vs 44.7%; *P* = .002) serving at the frontlines. Among these frontliners, 42.9% were doctors and nurses, 21.4% were allied health professionals and the remaining 35.7% were ancillary staff. Most doctors and nurses (70.8%) worked in hospitals, from different departments ranging from emergency department to surgical and medical departments (Table 1). Comparatively, there were lower proportions of allied health professionals (41.7%) and frontline ancillary staff (50.0%) who worked in hospitals (Tables 2 and 3). There was a wide range of occupations among the ancillary staff, including receptionist, porter and coordinator. Healthcare assistants and porters constituted more than one‐third (40.0%) of the frontline ancillary staff. Among those working at the back‐end, majority (81.3%) worked in hospitals, including 5 construction workers at hospital grounds. Majority of the back‐end HCWs were ancillary staff (59.4%), constituted primarily by technicians, maintenance workers and cleaners/housekeepers. Overall, ancillary staff constituted the largest proportion of HCWs (44.3%) infected with COVID‐19.

Most of the HCWs acquired COVID‐19 infection locally (81.8%). However, there was significantly more frontline HCWs among imported cases than the locally acquired cases (87.5% vs 58.3%, *P* = .042). Doctors, nurses and allied health professionals made up 68.8% of all imported cases, whilst ancillary staff made up 48.6% of all locally acquired cases (Table 4).

**TABLE 4:** Sources of infection and exposure of healthcare workers with COVID‐19

Job category	Total		Source of infection				Local cases only (n = 72)							
			Imported		Local		Family/household		Social interaction		Workplace		Unidentified	
	No.	%	No.	%	No.	%	No.	%	No.	%	No.	%	No.	%
Total	**88**	**100.0**	**16**	**100.0**	**72**	**100.0**	**20**	**27.8**	**11**	**15.3**	**12**	**16.7**	**29**	**40.3**
Frontline/frequent patient contact	**56**	**63.6**	**14**	**87.5**	**42**	**58.3**	**7**	**16.7**	**8**	**19.0**	**6**	**14.3**	**21**	**50.0**
Doctor	**14**	**15.9**	**4**	**25.0**	**10**	**13.9**	**1**	**10.0**	**1**	**10.0**	**1**	**10.0**	**7**	**70.0**
Nurse	**10**	**11.4**	**2**	**12.5**	**8**	**11.1**	**3**	**37.5**	**1**	**12.5**	**0**	**0.0**	**4**	**50.0**
Allied health	**12**	**13.6**	**5**	**31.3**	**7**	**9.7**	**0**	**0.0**	**3**	**42.9**	**1**	**14.3**	**3**	**42.9**
Medical social worker	*3*	*3.4*	*0*	*0.0*	*3*	*4.2*	*0*	*0.0*	*3*	*100.0*	*0*	*0.0*	*0*	*0.0*
Physiotherapist/speech therapist	*3*	*3.4*	*2*	*12.5*	*1*	*1.4*	*0*	*0.0*	*0*	*0.0*	*0*	*0.0*	*1*	*100.0*
Psychologist/psychotherapist	*2*	*2.3*	*2*	*12.5*	*0*	*0.0*	*0*	*‐*	*0*	*‐*	*0*	*‐*	*0*	*‐*
Traditional Chinese Medicine physician	*2*	*2.3*	*0*	*0.0*	*2*	*2.8*	*0*	*0.0*	*0*	*0.0*	*0*	*0.0*	*2*	*100.0*
Radiographer	*1*	*1.1*	*1*	*6.3*	*0*	*0.0*	*0*	*‐*	*0*	*‐*	*0*	*‐*	*0*	*‐*
Pharmacist	*1*	*1.1*	*0*	*0.0*	*1*	*1.4*	*0*	*0.0*	*0*	*0.0*	*1*	*100.0*	*0*	*0.0*
Ancillary staff	**20**	**22.7**	**3**	**18.8**	**17**	**23.6**	**3**	**17.6**	**3**	**17.6**	**4**	**23.5**	**7**	**41.2**
Healthcare assistant/attendant	*4*	*4.5*	*1*	*6.3*	*3*	*4.2*	*2*	*66.7*	*1*	*33.3*	*0*	*0.0*	*0*	*0.0*
Porter	*4*	*4.5*	*2*	*12.5*	*2*	*2.8*	*0*	*0.0*	*0*	*0.0*	*1*	*50.0*	*1*	*50.0*
Clinic assistant	*2*	*2.3*	*0*	*0.0*	*2*	*2.8*	*0*	*0.0*	*0*	*0.0*	*2*	*100.0*	*0*	*0.0*
Patient service associate	*2*	*2.3*	*0*	*0.0*	*2*	*2.8*	*0*	*0.0*	*0*	*0.0*	*0*	*0.0*	*2*	*100.0*
Coordinator	*2*	*2.3*	*0*	*0.0*	*2*	*2.8*	*0*	*0.0*	*1*	*50.0*	*0*	*0.0*	*1*	*50.0*
Dental assistant	*1*	*1.1*	*0*	*0.0*	*1*	*1.4*	*0*	*0.0*	*0*	*0.0*	*0*	*0.0*	*1*	*100.0*
Nursing aide	*1*	*1.1*	*0*	*0.0*	*1*	*1.4*	*0*	*0.0*	*0*	*0.0*	*0*	*0.0*	*1*	*100.0*
Receptionist	*1*	*1.1*	*0*	*0.0*	*1*	*1.4*	*1*	*100.0*	*0*	*0.0*	*0*	*0.0*	*0*	*0.0*
Patient therapy associate	*1*	*1.1*	*0*	*0.0*	*1*	*1.4*	*0*	*0.0*	*0*	*0.0*	*0*	*0.0*	*1*	*100.0*
Health screening executive	*1*	*1.1*	*0*	*0.0*	*1*	*1.4*	*0*	*0.0*	*0*	*0.0*	*1*	*100.0*	*0*	*0.0*
Clinical specialist	*1*	*1.1*	*0*	*0.0*	*1*	*1.4*	*0*	*0.0*	*1*	*100.0*	*0*	*0.0*	*0*	*0.0*
Back‐end/minimal patient contact	**32**	**36.4**	**2**	**12.5**	**30**	**41.7**	**13**	**43.3**	**3**	**10.0**	**6**	**20.0**	**8**	**26.7**
Administrative staff	**8**	**9.1**	**1**	**6.3**	**7**	**9.7**	**1**	**14.3**	**2**	**28.6**	**1**	**14.3**	**3**	**42.9**
Administrator	*3*	*3.4*	*0*	*0.0*	*3*	*4.2*	*1*	*33.3*	*1*	*33.3*	*0*	*0.0*	*1*	*33.3*
Manager	*2*	*2.3*	*0*	*0.0*	*2*	*2.8*	*0*	*0.0*	*0*	*0.0*	*1*	*50.0*	*1*	*50.0*
Director	*1*	*1.1*	*0*	*0.0*	*1*	*1.4*	*0*	*0.0*	*0*	*0.0*	*0*	*0.0*	*1*	*100.0*
Researcher	*1*	*1.1*	*1*	*6.3*	*0*	*0.0*	*0*	*‐*	*0*	*‐*	*0*	*‐*	*0*	*‐*
Information technology	*1*	*1.1*	*0*	*0.0*	*1*	*1.4*	*0*	*0.0*	*1*	*100.0*	*0*	*0.0*	*0*	*0.0*
Ancillary staff	**19**	**21.6**	**1**	**6.3**	**18**	**25.0**	**9**	**50.0**	**1**	**5.6**	**3**	**16.7**	**5**	**27.8**
Technician	*10*	*11.4*	*0*	*0.0*	*10*	*13.9*	*5*	*50.0*	*1*	*10.0*	*2*	*20.0*	*2*	*20.0*
Maintenance worker	*4*	*4.5*	*0*	*0.0*	*4*	*5.6*	*2*	*50.0*	*0*	*0.0*	*1*	*25.0*	*1*	*25.0*
Cleaner or housekeeper	*3*	*3.4*	*1*	*6.3*	*2*	*2.8*	*1*	*50.0*	*0*	*0.0*	*0*	*0.0*	*1*	*50.0*
Medicine despatcher	*1*	*1.1*	*0*	*0.0*	*1*	*1.4*	*0*	*0.0*	*0*	*0.0*	*0*	*0.0*	*1*	*100.0*
Painter	*1*	*1.1*	*0*	*0.0*	*1*	*1.4*	*1*	*100.0*	*0*	*0.0*	*0*	*0.0*	*0*	*0.0*
Construction worker	**5**	**5.7**	**0**	**0.0**	**5**	**6.9**	**3**	**60.0**	**0**	**0.0**	**2**	**40.0**	**0**	**0.0**

The locally acquired cases were epidemiologically linked to exposures from family/household (27.8%), workplace (16.7%) and social interaction (15.3%), whilst 40.3% were undetermined. Exposure sources among the HCWs varied between frontline and back‐end HCWs. Among the frontline HCWs, 50.0% had no identifiable source. For those with known sources, social interaction (19.0%) was most common, followed by family/household (16.7%) and workplace (14.3%) exposures. Most of the HCWs with workplace exposure (5 cases, 83.3%) were ever exposed to infected patients and the remaining one HCW to other infected HCWs. Comparatively, family/household exposure was most common among back‐end HCWs (43.3%) with a smaller proportion (26.7%) having no identifiable source (Table 4). All back‐end staff with workplace exposure had ever exposed to other infected HCWs.

During the study period, 14 HCWs (15.9%) were epidemiologically linked to clustered infections in foreign worker dormitories. All of them were back‐end ancillary staff or construction workers. Another five HCWs (5.7%) were linked to two religious gathering events. Lastly, three (3.4%) were linked to a clustered infection in an old‐folks home and another three (3.4%) to a shopping mall.

All HCWs were discharged well from hospital: 28.4% directly to home, 68.2% to community facilities and the remaining 3.4% to community hospitals. Three HCWs (3.4%) were ever admitted to the intensive care unit (ICU) during the study period and required oxygen supplementation. They were local residents aged between 59 and 61 years, and two were male. ICU care was started 3‐5 days after hospital admission, and their median length of stay in ICU was 8 days (3‐12 days). There was no mortality among all infected HCWs.

## 4 DISCUSSION

During the first 86 days since the detection of Singapore's first COVID‐19 case, only 1.7% of cases were HCWs. This was lower than the 2.4%‐11% reported in the literature.^13^, ^14^, ^15^ China first reported cases of COVID‐19 on 31 December 2019. Soon after, Singapore health officials monitored its development closely and quickly stepped up control measures, including crisis response preparations and heightened vigilance of the healthcare system. After the SARS outbreak in 2003, Singapore built and commissioned the National Centre for Infectious Diseases (NCID) in 2019. There are well‐established infection control and preventive strategies such as enforced use of PPE, regular temperature monitoring and monitoring clusters of sick hospital staff using the web‐based staff surveillance systems^11^ to minimize transmission risk in hospital settings should an outbreak occurred again. These infection control and preventive strategies were further enhanced at the start of the COVID‐19 outbreak. We postulated that these strategies had contributed to the low infection rate among HCWs in Singapore, as literature^16^, ^17^ had reported significant reduction in disease transmission in healthcare settings after staff protection measures were instituted.

Majority of our HCWs was young with median age of 35 years, lower than 39‐49 years reported in the literature.^14^, ^18^, ^19^ The disparities could be due to the varying definitions of HCWs adopted in different studies, with many studies including only personnel who work in healthcare settings.^14^, ^18^ Our study also included HCWs who worked in non‐healthcare institutions, as well as technicians and construction workers involved in hospital construction and renovation works within hospital campus. It was unclear whether these workers had been included in the literature.^14^, ^18^, ^19^ They were primarily foreign workers who were significantly younger than the local HCWs and originated from India. Correspondingly, Indians were disproportionately higher relative to the national ethnic distribution in Singapore.

The first HCW to be detected with COVID‐19 in Singapore on 13 February was a doctor who acquired the infection locally, but the exposure source was unidentified. Subsequently, another 13 doctors working at hospitals, national specialty centres, primary care clinics and other institutions were diagnosed between 19 March and 13 April 2020 and all had favourable outcomes. A study^20^ that included 198 deaths among doctors worldwide reported that the median age of the death cases was 66 years; approximately 40% of them were from emergency department or worked as general practitioners. Lack of PPE was reported to be the common contributor to the cause of death.^20^ In our study, the favourable outcomes among doctors could be accounted by their younger age profile (median: 39.5 years; range: 25‐67 years), use of PPE at workplace, early disease detection as part of enhanced surveillance efforts and the corresponding early intervention. Whilst frontline medical staff, such as doctors and nurses, is at higher risk of infection due to increased exposure to SARS‐CoV‐2, ancillary staff constituted the largest proportion of HCWs infected with COVID‐19 in Singapore. Those who worked at the back‐end accounted for more than one‐third of all cases, and many had acquired infection in the community or from family/household members. Of the three HCWs admitted to ICU, two served at the back‐end. There should therefore be adequate safe management measures at the workplaces to reduce the chance of spread to fellow colleagues in the hospital or clinic.

Outside of work, HCWs remained vulnerable to SARS‐CoV‐2 at home or at other social events when not in PPE, such as having meals with family or colleagues, or meetings at workplace as transmission could be facilitated at social gatherings.^21^, ^22^ In this study, family/household was the most common exposure source, which corroborated previous findings^23^ on the highest family/household transmission risks resulted from prolonged close contacts. Our study showed that the exposure from family/household was more prevalent among the back‐end HCWs with disproportionately more foreigners. The earliest infection that occurred to HCWs who stayed in the foreign worker dormitories was 31 March. Thereafter, another 13 foreign HCWs from five other dormitories were also infected between 4 April and 9 April. Workers from different workplaces were staying in the same dormitories and shared common facilities. Many of them had substantial household interactions with roommates or social mingling in their dormitories where social distancing measures were difficult to be maintained. Hence, there is a need for HCW to observe infection control and preventive measures beyond healthcare settings. Personal protection, such as the use of PPE at work, regular temperature monitoring, good personal hygiene and safe distancing, need to be adhered to at all times.

This study adds to the limited literature on the range of occupations and job nature of HCWs infected with COVID‐19 and sheds lights on workers who are exposed to infectious disease due to work activities. This is an important first step for policymakers in infectious disease risk management prior to transmission containment efforts. Apart from the conventional medical personnel such as doctors, nurses and allied health professionals, this study highlighted a wide range of occupations among HCWs ranging from cleaners and construction workers to alternative medicine practitioners (eg Traditional Chinese Medicine physicians). Regardless of whether the HCW serves at the frontline or back‐end, they are at risk of infection. This study also included HCWs working in non‐healthcare settings such as community services (eg old‐folks home and charitable organizations) as they may be serving vulnerable groups such as the elderly or poor and needy individuals. In Singapore, clustered infections were detected beyond healthcare settings such as old‐folks home and dormitories of foreign HCWs. Quantifying the number of these HCWs and understanding their profiles allow for the management of COVID‐19 and health risk response beyond health care to prevent disease transmission to vulnerable groups they serve or have regular contact with.

This study has some limitations. Data of occupation and workplace were collected by trained contact tracing personnel, but some data may be missing and the proportion of HCWs could have been under‐estimated. Recall bias may affect the accuracy of self‐reported symptom onset date and the activity maps. There is increasing evidence^24^ of pre‐symptomatic transmission. Identifying probable infector using chronological symptom onset dates could mis‐specify the exposure source. There are a high proportion of cases with unidentifiable exposure source, due partially to the asymptomatic cases who did not have symptom onset date. However, they were unlikely to be linked to workplace exposure at hospital or clinics, as close contacts at the workplaces, including colleagues and patients, were tested negative for COVID‐19. In Singapore, the transmission of SARS‐CoV‐2 during the initial phases of COVID‐19 outbreak was primarily at community instead of at healthcare settings.^22^ Thus, we postulated that majority of cases with unidentifiable exposure source probably acquired the virus in the community during the early phase of local spread.

In conclusion, HCWs accounted for a small proportion of all COVID‐19 cases in Singapore with favourable outcomes, signalling that the existing infective control practices and measures at healthcare settings in Singapore are adequate. All HCWs, regardless of their occupation, workplace settings and working at frontline or back‐end, are important human resources for medical outbreak response. To ensure sustainability of our healthcare system, all HCWs need to be protected to prevent spread to one another and to patients causing iatrogenic outbreak. The possibility of transmission resulting from family or household exposures and social interactions highlighted the need to maintain strict vigilance and precautionary measures even beyond the healthcare environment.

## AUTHOR CONTRIBUTION

**Lai Yin Wong**: Conceptualization (equal); Formal analysis (equal); Methodology (equal); Writing‐original draft (equal); Writing‐review & editing (equal). **Aidan Lyanzhiang Tan**: Writing‐review & editing (equal). **Yee Sin Leo**: Writing‐review & editing (equal). **Vernon Lee**: Writing‐review & editing (equal). **Matthias Paul Han Sim Toh**: Conceptualization (equal); Supervision (equal); Writing‐review & editing (equal).

**CONFLICT OF INTERESTS**

All authors report no conflicts of interest relevant to this article.

## Data Availability

The data that support the findings of this study are available from the corresponding author upon reasonable request.
